# A CT-based radiomics nomogram for the preoperative prediction of perineural invasion in pancreatic ductal adenocarcinoma

**DOI:** 10.3389/fonc.2025.1525835

**Published:** 2025-03-04

**Authors:** Yan Deng, Haopeng Yu, Xiuping Duan, Li Liu, Zixing Huang, Bin Song

**Affiliations:** ^1^ Department of Radiology, Functional and Molecular Imaging Key Laboratory of Sichuan Province, West China Hospital, Sichuan University, Chengdu, China; ^2^ Department of Radiology, Sichuan Key Laboratory of Medical Imaging, Affiliated Hospital of North Sichuan Medical College, Nanchong, Sichuan, China; ^3^ Department of Radiology, Sanya People’s Hospital, Sanya, Hainan, China

**Keywords:** pancreatic ductal adenocarcinoma, perineural invasion, computed tomography, radiomics, nomogram

## Abstract

**Purpose:**

To develop a nomogram based on CT radiomics features for preoperative prediction of perineural invasion (PNI) in pancreatic ductal adenocarcinoma (PDAC) patients.

**Methods:**

A total of 217 patients with histologically confirmed PDAC were enrolled in this retrospective study. Radiomics features were extracted from the whole tumor. Univariate analysis, least absolute shrinkage and selection operator and logistic regression were applied for feature selection and radiomics model construction. Finally, a nomogram combining the radiomics score (Rad-score) and clinical characteristics was established. Receiver operating characteristic curve analysis, calibration curve analysis and decision curve analysis (DCA) were used to evaluate the predictive performance of the nomogram.

**Results:**

According to multivariate analysis, CT features, including the radiologists evaluated PNI status based on CECT (CTPNI) (OR=1.971 [95% CI: 1.165, 3.332], P=0.01), the lymph node status determined on CECT (CTLN) (OR=2.506 [95%: 1.416, 4.333], P=0.001) and the Rad-score (OR=3.666 [95% CI: 2.069, 6.494], P<0.001), were significantly associated with PNI. The area under the receiver operating characteristic curve (AUC) for the nomogram combined with the Rad-score, CTLN and CTPNI achieved favorable discrimination of PNI status, with AUCs of 0.846 and 0.778 in the training and testing cohorts, respectively, which were superior to those of the Rad-score (AUC of 0.720 in the training cohort and 0.640 in the testing cohort) and CTPNI (AUC of 0.610 in the training cohort and 0.675 in the testing cohort). The calibration plot and decision curve showed good results.

**Conclusion:**

The CT-based radiomics nomogram has the potential to accurately predict PNI in patients with PDAC.

## Introduction

Pancreatic ductal adenocarcinoma (PDAC) is the third leading cause of cancer-related death, and 66,440 new cases and 51,750 new deaths are estimated to occur in 2024 (1). Radical resection is the only effective means for treatment, but fewer than 20% of patients are able to undergo surgery at the time of diagnosis, and early recurrence and metastasis frequently occur after radical resection (2).

PDAC is characterized by perineural growth, and its incidence is 43.2%-100% (3). Perineural invasion (PNI) is related to the dissemination and metastasis of PDAC and is an independent risk factor for patient prognosis (4–6). A previous study showed that patients who received neoadjuvant therapy had a significantly lower PNI than did those who did not receive neoadjuvant therapy (7). Felsenstein et al. demonstrated that adjuvant chemotherapy improved the prognosis in patients with PNI-positive PDAC but not in those with PNI-negative disease (8). In addition, the PNI status affects whether the Heidelberg procedure is performed (9). The assessment of PNI currently relies on histopathology following surgery; however, preoperative knowledge of PNI status holds clinical significance because it has the potential to aid clinicians in identifying high-risk categories beforehand, formulating personalized treatment plans, and ultimately improving patient outcomes.

Contrast-enhanced CT (CECT) is the first-line imaging method for the diagnosis, staging and evaluation of the therapeutic effect of PDAC (10) and has been applied for operatively evaluating PNI in PDAC (11). Previous studies (12–14) have evaluated PNI of PDAC via qualitative methods and established criteria for CECT; however, few study objectively evaluated PNI on CECT. Guo et al. (15) established a quantitative method based on the minimum distance between the tumor boundary and adjacent arteries, but the tumor boundary is difficult to define and may affect the measurement results.

Radiomics can noninvasively and rapidly obtain diagnostic, prognostic and treatment information from medical images to support clinical decision-making. This information can be used as a complementary tool to verify clinical and imaging results (16–18). Radiomics has been applied to evaluate lymph node (LN) metastasis and assess the prognosis of PDAC patients (19–21). Several CT/MRI-based radiomics models for predicting PNI have been introduced for rectal cancer and gastric cancer patients, and they have achieved satisfactory results (22, 23). However, few studies evaluated PNI preoperatively in patients with PDAC based on CT radiomics.

The aim of this study was to develop and validate a nomogram based on CT radiomics features and clinical characteristics for the preoperative prediction of PNI in PDAC patients.

## Materials and methods

This retrospective study was approved by the institutional review board at West China Hospital, Sichuan, China (IRB number: 2023-0003), and the requirement for written informed consent was waived. It performed in accordance with Declaration of Helsinki.

### Patients

**Figure 1:**
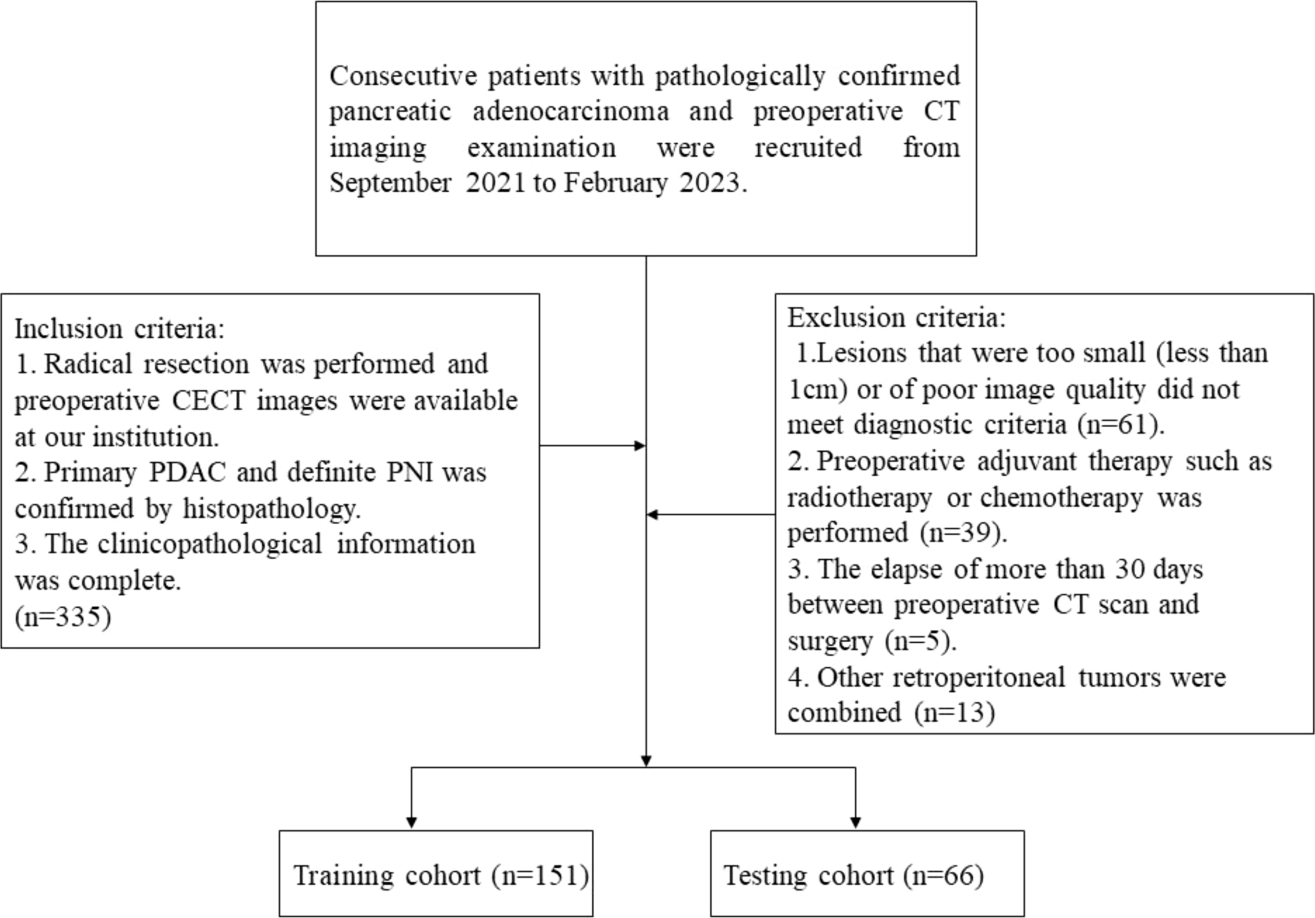
Flow chart of patient recruitment. CECT, contrast-enhanced computed tomography; PDAC, Pancreatic ductal adenocarcinoma; PNI, Perineural invasion.

A total of 335 patients with PDAC who underwent CT at our hospital between September 2021 and February 2023 were enrolled in this retrospective study. The inclusion criteria were as follows: (1) underwent radical resection, and preoperative CECT images were available at our institution, (2) primary PDAC and definite PNI were confirmed by histopathology, and (3) complete clinicopathological information was available. The exclusion criteria were as follows: (1) lesions that were too small (less than 1 cm) or of poor image quality that did not meet diagnostic criteria, (2) preoperative neoadjuvant therapy such as radiotherapy or chemotherapy, (3) more than 30 days between the preoperative CT scan and surgery, or (4) other retroperitoneal tumors. Finally, 217 patients with PDAC were enrolled (99 PNI-negative patients and 118 PNI-positive patients), and the patients were randomly divided into a training cohort (n = 151) and a validation cohort (n = 66) at a ratio of 7:3 (**Figure 1**).

Demographic information, including age and sex, was collected. The carbohydrate antigen 19-9 (CA19-9) and carcinoembryonic antigen (CEA) levels before surgery were recorded.

### Pathological PNI diagnosis

PNI is defined as a tumor located near a nerve, with tumor cells located in at least 33% of the nerve perimeter or in any of the three layers of the nerve sheath (24–26).

### CT image acquisition

The abdominal CT scanning parameters and contrast agents used are described in detail in **Supplementary A1** in **Supplementary Data Sheet 1**.

### CT image analysis

Two radiologists with six and eight years of experience in abdominal imaging who were blinded to the pathologic details reviewed the CT images and evaluated the following features: radiologists evaluated PNI status based on CECT (CTPNI), lymph node status determined on CECT (CTLN), location and size of the tumor, and dilatation of the common bile duct and the main pancreatic duct. Discrepancies between observers were resolved by consensus, and further analysis was performed using consensus interpretation. Interagreement between the two reviewers was evaluated by calculating the intraclass correlation coefficient (ICC) for continuous variables and Cohen’s kappa value for categorical variables.

CTPNI was defined as the disappearance of the peripancreatic fat space or peripancreatic vascular space (including the common hepatic artery, superior mesenteric artery, superior mesenteric vein, celiac artery and splenic vessels) or the appearance of ribbon-like, reticular soft tissue density shadows or irregular mass shadows (27).If any of the following conditions were met, the CTLN was evaluated as positive: the short diameter of the LN was more than 10 mm, the density was uneven, the enhancement was uneven, internal necrosis occurred, the LN was fused, the boundary of the LN was unclear, or the LN invaded adjacent organs or blood vessels (28).

The location and size of the tumor were recorded based on preoperative CT. The location of the tumor was defined as the head on the right side, the neck in front, or the body or tail on the left side according to the confluence of the portal vein and the superior mesenteric vein. The size of the tumor was measured at the axial level according to the largest cross section of the lesion. Tumor size was calculated as the mean of two measurements for further analysis.

A common bile duct (CBD) diameter greater than 10 mm and a main pancreatic duct (MPD) diameter greater than 2 mm were defined as dilated (29).

### Tumor segmentation

Two experienced radiologists in abdominal imaging (with six and eight years of experience from Reader 1 and Reader 2, respectively) manually and independently and blindly sketched the lesion slice by slice along the edge to the results from 30 randomly selected patients based on the arterial and portal venous images of CECT, avoiding the CBD and vessels. Reader 1 sketched the lesion twice, with an interval of more than 1 week, for calculating intra-agreement. Reader 1 and Reader 2 blindly and independently delineated the lesion to measure the interagreement. Then, the sketch of all patients was completed by reader 1. This process was implemented on the open source software IBEX (β1.0, http://bit.ly/IBEX_MDAnderson), which runs on MATLAB 2013a. Due to the variability problems caused by voxel size and gray level dependence, it is unrealistic that all radiomics features achieve satisfactory agreement. The ICC was used to assess intraobserver and interobserver agreement. An ICC greater than 0.75 was considered to indicate good consistency.

### Radiomics extraction and selection

**Figure 2:**
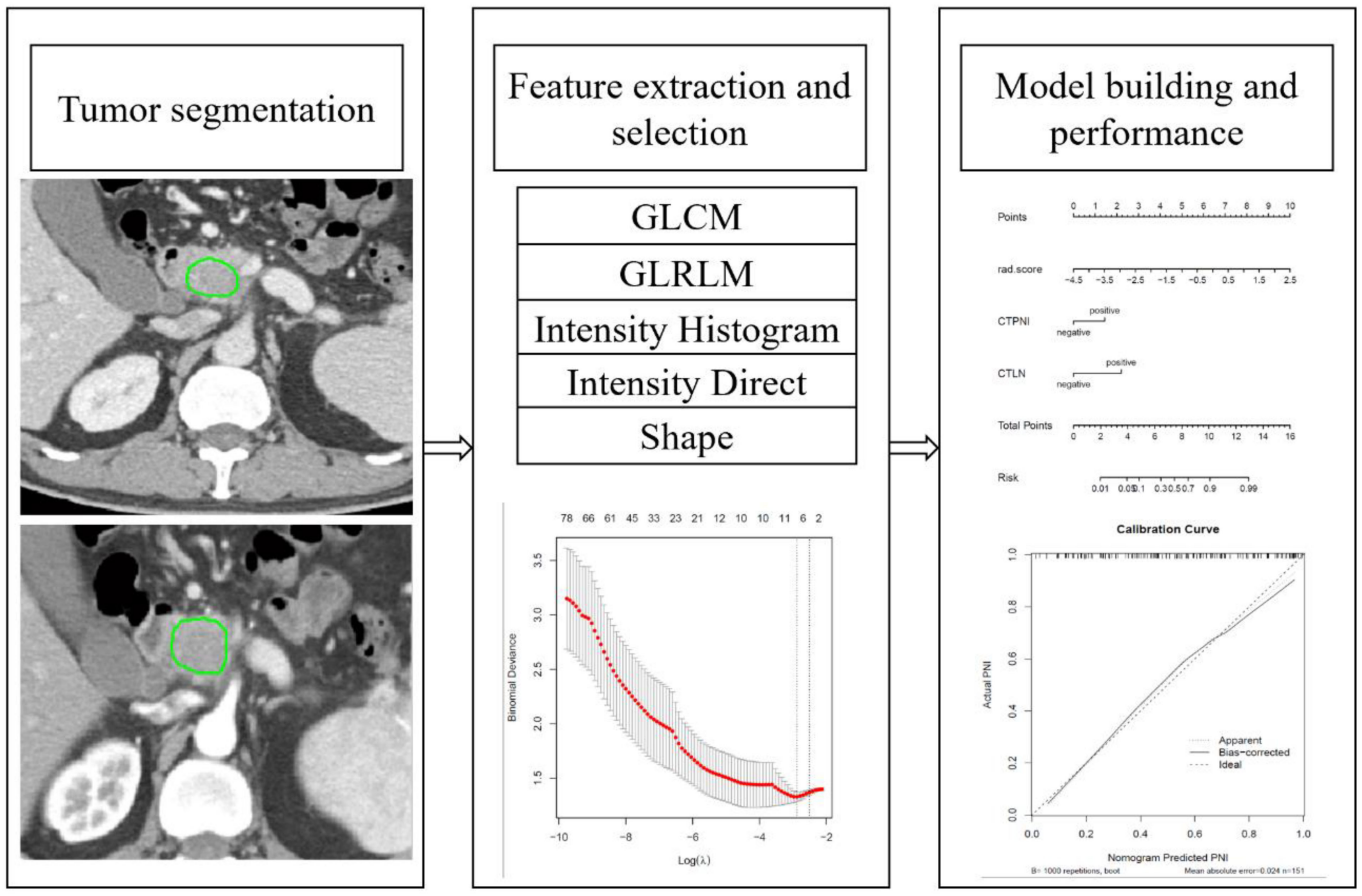
Radiomics workflow. GLCM: Gray-level cooccurrences matrix. GLRLM: Gray-level run-length matrix.

The gray level cooccurrence matrix (GLCM), gray level runlength matrix (GLRLM), intensity histogram (IH), intensity direct (ID) and shape feature groups were extracted from IBEX. Based on the arterial and portal venous phase, 404 radiomics features were extracted respectively and a total of 808 radiomics features were extracted for analysis. The detail of Feature extraction in **Supplementary S1** in **Supplementary Data Sheet 1**. Resampling was applied for preprocessing to eliminate images with different scanning parameters and slice thicknesses (30). The Z score was used to eliminate the effect of the data dimension. The radiomics workflow is shown in **Figure 2**. The detail of preprocessing methods for the image and data in **Supplementary S2** in **Supplementary Data Sheet 1**.

The features extracted from arterial phase and portal phase were combined for dimension reduction analysis. Two steps were adopted for reducing the dimensions and identifying robust radiomics features in the training cohort. Univariate analysis with an independent samples t test or the Mann-Whitney U test was first applied to select potentially important features. Subsequently, the least absolute shrinkage and selection operator (LASSO) method was applied using tenfold cross-validation for feature selection. Lambda was selected according to the 1-standard error of the minimum criterion (1-SE criterion, a simpler model). The selected optimal radiomics features were weighted by their respective coefficients and a linear combination to obtain the corresponding radiomics score (Rad-score) in the training and testing cohorts.

### Nomogram construction

The significantly different features were used to construct a nomogram for both the training and testing cohorts. The area under the receiver operating characteristic (ROC) curve (AUC), accuracy, sensitivity and specificity were calculated to assess the performance of the nomogram model. A calibration curve was used to evaluate the agreement between the predicted probability of PNI and the actual probability of PNI. The clinical utility of the model was evaluated by a decisive curve.

### Statistical analysis

Statistical analyses of clinical characteristics were conducted with SPSS (statistics 26). The remaining statistical analyses were implemented in R (version 4.3.1, https://www.r-project.org/). A significant difference was considered at P<0.05. Categorical variables were analyzed using the chi-square test or Fisher’s exact test. Continuous variables were analyzed by the independent samples t test or the Mann-Whitney U test, depending on the type of data distribution. Univariate and multivariate logistic regression analysis were used to identify independent predictors that were significantly associated with PNI.

The “glmnet” PDACkage was used for LASSO regression analysis. The “rms” PDACkage was applied for nomogram construction and calibration curve plotting. The “pROC” PDACkage and “dca. R” were used for the ROC curve and decision curve plot, respectively.

## Results

### Clinical characteristics

**Table 1** showed the clinical data for patients in the training cohort and testing cohort. The LN status and tumor histopathological grade were significantly different between the two groups in the training cohort (P<0.05), while there was no significant difference in the testing cohort (P>0.05). The CTLN and CTPNI were significantly different in both the training and testing cohorts (P<0.05).

**Table 1:** The patients’ characteristics in the training and testing cohorts.

Characteristics	Training cohort		P value	Testing cohort		P value
	PNI (+) (n=82)	PNI (-) (n=69)		PNI (+) (n=36)	PNI (-) (n=30)	
Age (y)	60.1±11.0	60.4±9.2	0.451	59.3±11.3	57.9±10.1	0.578
Sex			0.225			0.451
Male	48	47		22	21	
Female	34	22		14	9	
Interval between CT and surgery (d)	5 (3,8)	6 (3, 9)	0.401	4 (2,7)	4 (2, 6)	0.869
Location			0.903			0.794
Head	53	47		30	24	
Neck	4	3		3	2	
Body or tail	25	19		3	4	
Vascular			0.863			0.288
Negative	54	47		26	25	
Positive	28	22		10	5	
Margin			0.641			0.114
Negative	73	63		30	29	
Positive	9	6		6	1	
MPD			0.229			0.203
Negative	21	23		7	10	
Positive	61	46		29	20	
CBD			0.284			0.575
Negative	38	26		12	12	
Positive	44	43		24	18	
CTLN			<0.001*			0.034*
Negative	47	61		21	25	
Positive	35	8		15	5	
CTPNI			0.006*			0.004*
Negative	45	53		21	28	
Positive	37	16		15	2	
Size	2.50 (1.90, 4.05)	2.24 (1.84, 3.09)	0.155	2.55 (1.94, 3.19)	2.42 (2, 3)	0.981
CEA	3.60 (2.21, 5.57)	3.32 (2.27, 5.51)	0.977	3.12 (2.21, 7.4)	3.02 (1.72, 6.2)	0.747
CA19-9	270 (65.89, 795.5)	195. 45 (26.23, 577. 63)	0.375	308.25 (75.0, 699.55)	196.25 (61.15, 387.95)	0.245
Type of pancreatic surgery			0.935			0.979
Standard or extended pancreaticoduodenectomy	55	48		27	22	
Standard or extended distal pancreatectomy	20	16		7	6	
Total pancreatectomy	7	5		2	2	
LN			0.037*			0.389
Negative	42	47		19	19	
Positive	40	22		17	11	
Grade			0.049*			0.413
Well-differentiated	1	3		1	2	
Moderately differentiated	53	54		23	22	
Poorly differentiated	28	12		12	6	
Rad-score	0.52 (-0.06, 0.99)	-0.13 (-0.85, - 0.5)	<0.001*	0.46 (-0.14, 0.95)	-0.08 (-0.58, 0.6)	0.01*

* represents a statistically significant difference.

CTPNI: Radiologists evaluated the status of PNI based on CECT; CTLN: The lymph node status determined on CT; CA19-9: Carbohydrate antigen 19-9; CEA: Carcinoembryonic antigen; CBD: Common bile duct; LN: Lymph node; MPD: Main pancreatic duct; PNI: Perineural invasion; Rad-score: Radiomics score.

There was no significant difference between the training and validation cohorts in terms of the percentage of PNI-positive patients. There was no significant difference between the PNI-positive and PNI-negative groups in age, sex, CEA, CA19-9, tumor size or location, CBD or MPD dilation status, or vascular invasion or margin status in either the training or testing cohort. The ICC of the tumor size was 0.936, indicating good consistency. The kappa values of the CTPNI and CTLN were 0.723 and 0.741, respectively, with moderate consistency.

According to the univariate analysis of the training cohort, the CTPNI and CTLN were significantly associated with PNI (**Table 2**). According to multivariate analysis, CT features, including the CTPNI positive (OR=1.971 [95% confidence interval [CI]: 1.165, 3.332], P=0.01) and CTLN positive (OR=2.506 [95%: 1.416, 4.333], P=0.001), were significantly associated with PNI.

**Table 2:** Univariate and multivariate logistic regression analysis for PNI of PDAC.

Characteristics	Univariate Analysis		Multivariate Analysis	
	Odds Ratio (95% CI)	P value	Odds Ratio (95% CI)	P value
CTPNI (Positive vs Negative)	2.193 (1.277, 3.763)	0.006	1.971 (1.165, 3.332)	0.01
CTLN (Positive vs Negative)	2.596 (1.752, 3.847)	<0.001	2.506 (1.416, 4.333)	0.001
Tumor size	0.859 (0.696, 1.059)	0.155	NA	NA
MPD (Absence vs Presence)	0.689 (0.340, 1.393)	0.299	NA	NA
CBD (Absence vs Presence)	1.428 (0.744, 2.742)	0.284	NA	NA
CEA (continuous)	1.000 (0.992, 1.008)	0.977	NA	NA
CA19-9 (continuous)	1.000 (0.999, 1.001)	0.375	NA	NA
Rad-score (continuous)	2.718 (1.172, 4.316)	<0.001	3.666 (2.069, 6.494)	<0.001

CI, Confidence interval; CTPNI, Radiologists evaluated the status of PNI based on CECT; CTLN, The lymph node status determined on CT; Rad-score, Radiomics score.

### Radiomics feature selection and model construction

The mean ICCs for intraobserver agreement and interobserver agreement were 0.889 (range from 0.103 to 0.995) and 0.843 (range from 0.002-0.993), respectively (**Supplementary S3** and **Supplementary Figure S1** in **Supplementary Data Sheet 1**). Ninety radiomics features were excluded because of intraobserver agreement, and 141 radiomics features were excluded because of interobserver agreement. For features with suboptimal agreement, 68 intraobserver-excluded features were included among 141 interobserver-excluded features. Ultimately, 163 radiomics features were excluded due to inferior reproducibility, and the remaining 645 radiomics features were used for the next analysis.

After univariate analysis, 91 radiomics features were significantly different between the PNI-positive and PNI-negative groups in the training cohort. The LASSO regression method with 10-fold cross-validation was applied for the remaining features selected, and 8 optimized features were chosen for constructing the model (**Supplementary S4** in **Supplementary Data Sheet 1**). The selected radiomics features were quantitatively integrated into the Rad-score in the training and testing cohorts. According to the univariate and multivariate logistic regression analysis, the Rad-score (OR=3.666 [95% CI: 2.069, 6.494], P<0.001) was significantly related to PNI.

### Nomogram construction

**Figure 3:**
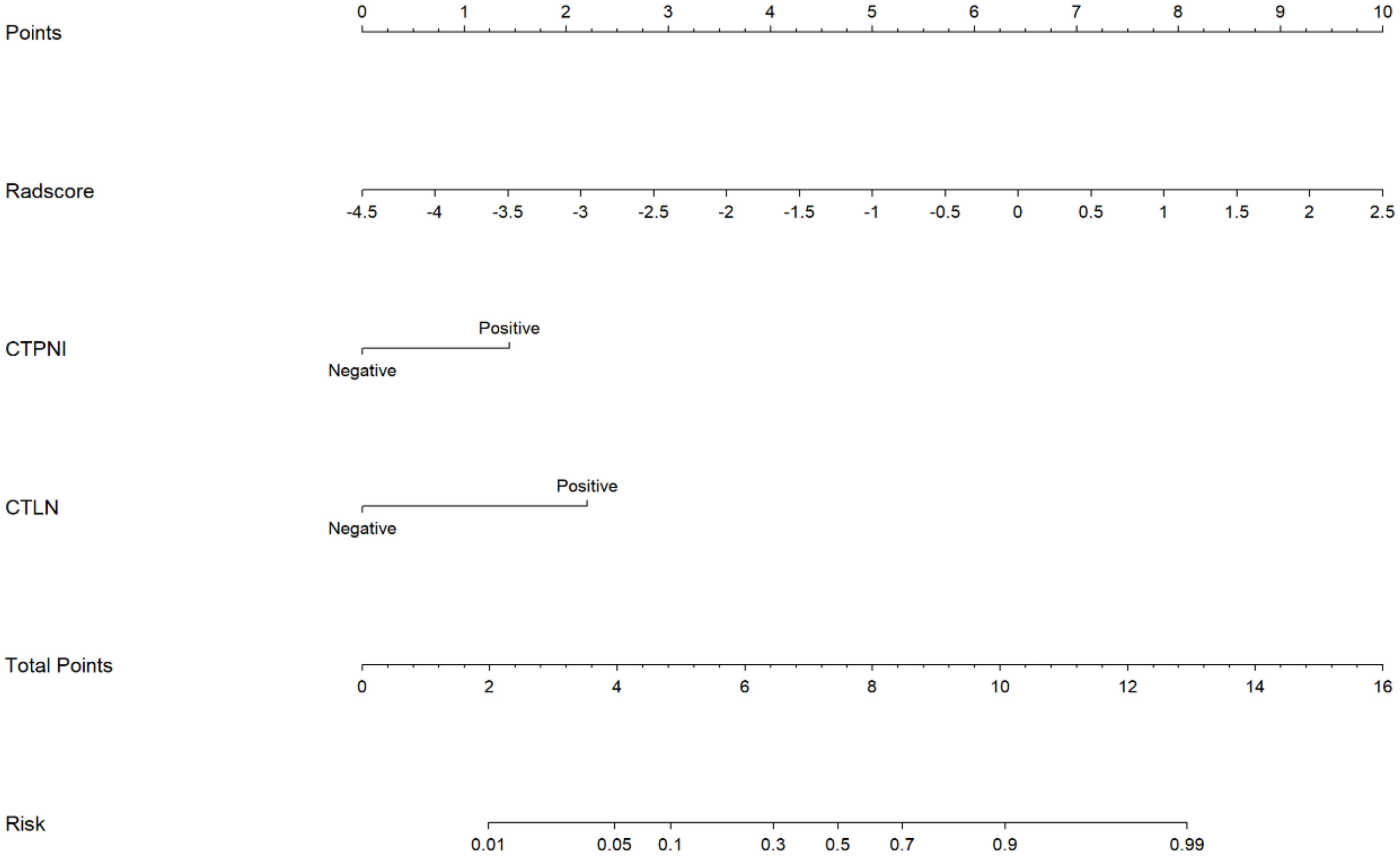
The nomogram for preoperatively evaluating the PNI status of PDAC patients. A nomogram combining the Rad-score, CTLN, and CTPNI for preoperative evaluation of PNI in PDAC patients. The Radscore, CTPNI and CTLN are summed to obtain the total points on the scale, and the risk of PNI in the PDAC is the corresponding number on the Risk axis. Radscore, Rad-score. CTLN; Lymph node status determined on CT; CTPNI, Radiologists evaluated the status of PNI based on CECT; PNI, Perineural invasion; PDAC, Pancreatic ductal adenocarcinoma.

**Figure 4:**
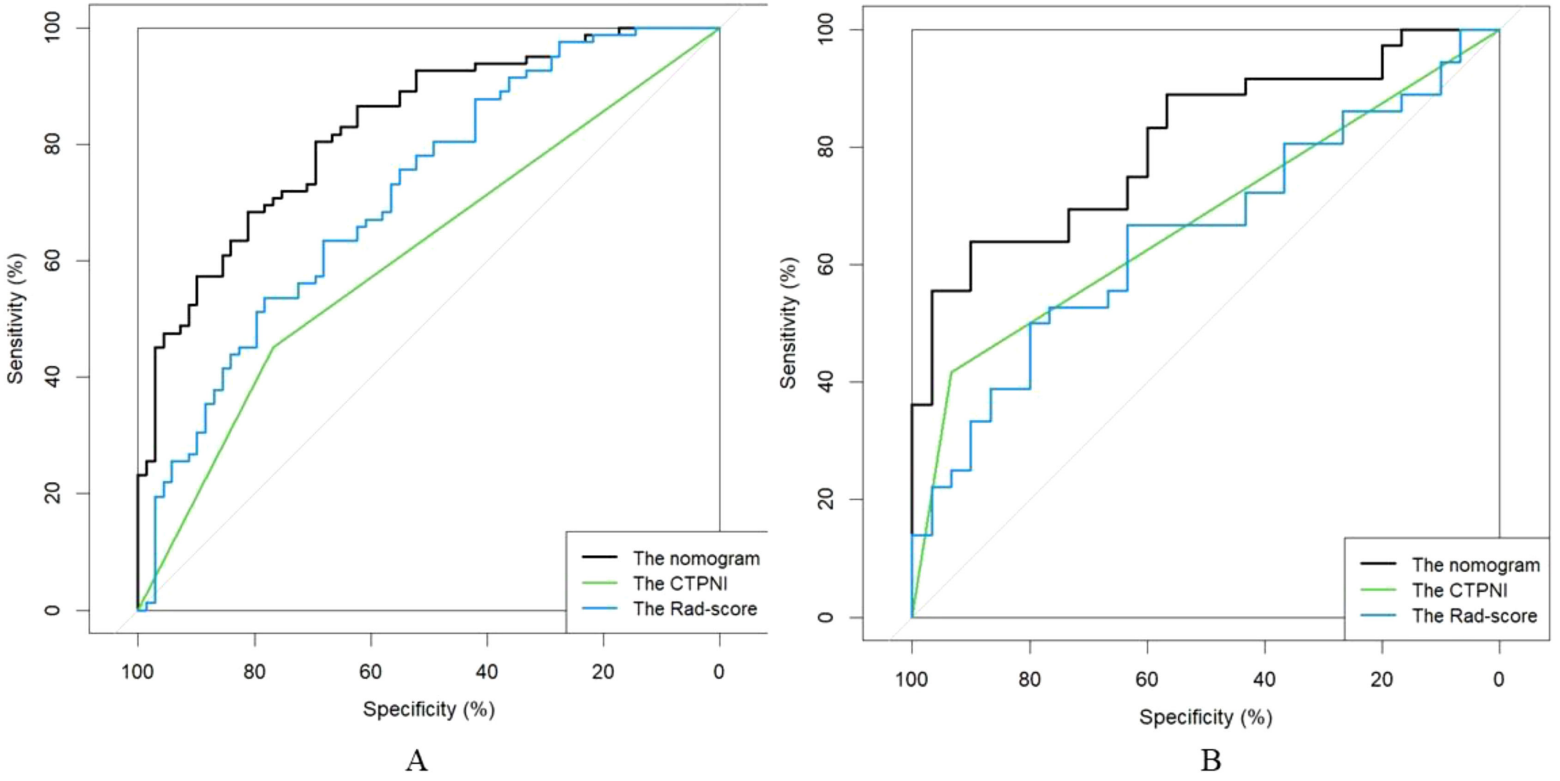
The ROC curve of the AUC comparison among the CTPNI model, radiomics model and Rad-clinical model. **(A)** The training cohort; **(B)** The testing cohort. ROC, Received operating characteristic; AUC, Area under the receiver operating characteristic curve; CTPNI, Radiologists evaluated the status of PNI based on CECT.

**Figure 5:**
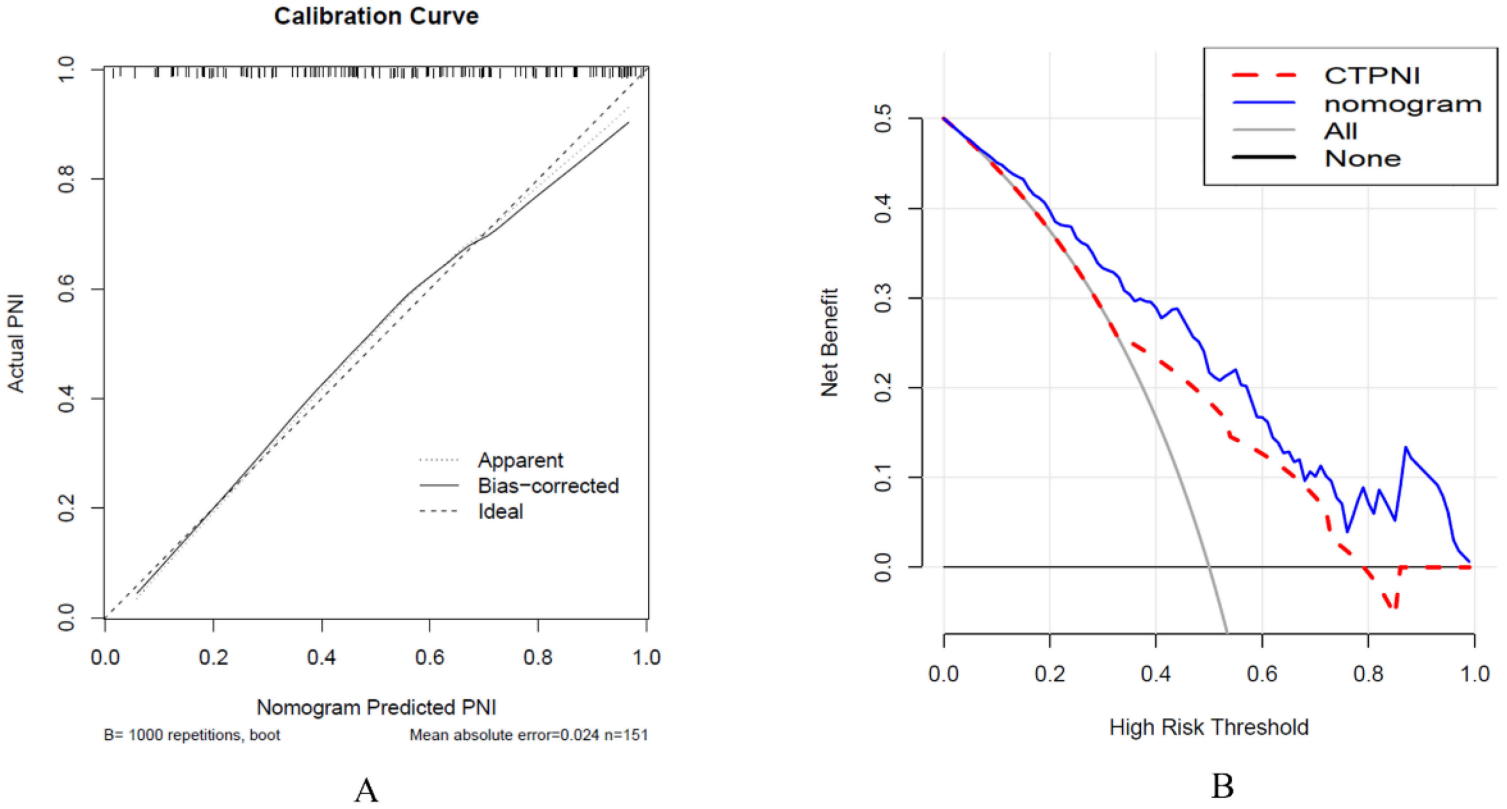
Calibration curve analysis was used to evaluate the nomogram performance **(A)**, and decision curve analysis was used **(B)**. **(A)** The x-axis represents the predicted PNI, and the y-axis represents the actual PNI. **(B)** The x-axis represents the threshold probability, and the y-axis represents the net benefit to the patient. PNI: Perineural invasion. CTPNI: Radiologists evaluated the status of PNI based on CECT.

**Figure 6:**
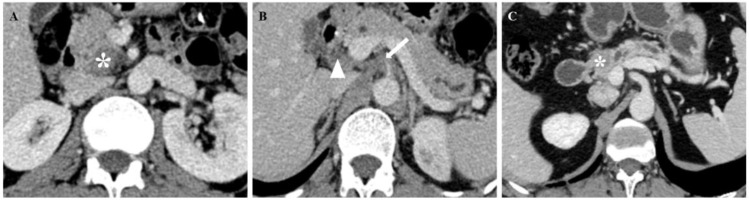
CT images of patients with PNI **(A, B)** and patients without PNI **(C)**. **(A, B)** A 67-year-old man with PDAC underwent preoperative contrast-enhanced axial abdominal CT, and the CTPNI (white arrow) and CTLN (white arrowhead) were positive. The lesion (* in A) was used to calculate a Rad-score of 1.11508, and the total score was 11.75. Based on the nomogram, a probability of PNI positivity greater than 0.95 and PNI positivity was confirmed by histopathology. **(C)** A 73-year-old man with PDAC who underwent preoperative contrast-enhanced axial abdominal CT, CTPNI and CTLN were negative, and the Rad-score was -1.89701, as calculated by delineating the lesion (* in C). The total score was 3.6, the probability of PNI positivity was less than 0.05, and PNI negativity was confirmed by histopathology. PNI, Perineural invasion; PDAC, Pancreatic ductal adenocarcinoma; CTLN, Lymph node status determined on CT; CTPNI, Radiologists evaluated the status of PNI based on CECT.

The Rad-score was independently associated with PNI. The AUC for the Rad-score in the training cohort (0.720, 95% CI: [0.639, 0.802]) was close to that in the testing cohort (0.640, 95% CI: 0.499, 0.781). The AUC of the CTPNI was 0.610 (95% CI: 0.536, 0.684) in the training cohort and 0.675 (95% CI: 0.582, 0.768) in the testing cohort. Using the Rad-score combined with the CTLN and CTPNI, a nomogram (**Figure 3**) for predicting PNI was constructed, and it achieved favorable performance both in the training cohort, with an AUC of 0.846 (95% CI: 0.785, 0.907), and in the testing cohort, with an AUC of 0.778 (95% CI: 0.666, 0.889). Comparing the AUCs of the nomogram model with those of the Rad-score and CTPNI through the DeLong test, the nomogram model achieved the best performance (P<0.05) (**Figure 4**). The diagnostic performance of the nomogram model, Rad-score and CTPNI in both the training and testing cohorts is summarized in **Table 3**. The calibration plot indicated that the predicted PNI based on the nomogram was consistent with the actual PNI (i.e., the status perineural invasion based on pathological result) probability (**Figure 5A**). The decision curve suggested that the nomogram model outperformed CTPNI at any threshold probability (**Figure 5B**). **Figure 6** shows examples of the clinical application of the nomogram.

**Table 3:** The performance of the training and testing cohorts.

Model		Accuracy	Sensitivity	Specificity	AUC (95% CI)
Training cohort	CTPNI model	0.596	0.451	0.768	0.610 (0.536, 0.684)
	Radiomics model	0.636	0.720	0.536	0.720 (0.639, 0.802)
	nomogram	0.781	0.890	0.768	0.846 (0.785, 0.907)
Testing cohort	CTPNI model	0.652	0.417	0.882	0.675 (0.582, 0.768)
	Radiomics model	0.636	0.722	0.533	0.640 (0.499, 0.781)
	nomogram	0.667	0.806	0.733	0.778 (0.666, 0.889)

AUC, Area under the receiver operating characteristic curve; CI, Confidence interval; Rad-clinical, The combined of CTLN, CTPNI and radiomics model.

## Discussion

Preoperative accurate evaluation of PNI in patients with PDAC affects the choice of appropriate treatment. Our retrospective study constructed a nomogram to preoperatively predict PNI based on the rad-score from the arterial and portal venous phases of CECT, CTLN and CTPNI. The nomogram could effectively predict the occurrence of PNI in both the training and testing groups. This study demonstrated that the nomogram was superior to the Rad-score and CTPNI for evaluating the occurrence of PNI.

The prevalence of PNI in PDAC patients is fairly high, varying from 42.3%-100% in previous reports (3), with an incidence of 54.4% in this study. Additionally, we found an interesting association between PNI status and LN status, and the PNI-positive group was more prone to LN metastasis. Previous studies have indicated that cancer cells grow along nerves in contact with LNs, indicating a complex link between LN metastasis and PNI (31, 32). There was also a statistically significant difference in the CTLNs. In addition, patients with PNI were more likely to have poor pathological differentiation. Poorly differentiated PDAC has more aggressive behavior, which is related to poor prognosis, PNI and poor differentiation and reflects the malignant biological behavior of PDAC. We did not find significant differences in tumor size; dilation status of the CBD or MPD; CEA or CA19-9 levels; or resection margin status between the PNI-positive and PNI-negative groups. The relationship between PNI and tumor size is controversial. Crippa et al. reported that the incidence of PNI increased with tumor size (33). However, Patel et al. suggested that no evidence was found between tumor size and PNI (11). PNI occurs early in PDAC, and even tumors less than 2 cm may develop PNI (34). This may be related to the greater probability of PNI due to tumor growth beyond the pancreas, while tumors within the pancreas generally do not develop PNI even if the tumor is large, based on the anatomical structure (35). These controversial results suggest that the relationship between tumor size and PNI needs further investigation. CBD and MPD dilatation caused by obstruction may not be associated with PNI. CEA and CA19-9 are nonspecific in PDAC and may be abnormal in a variety of diseases, which may account for the lack of differences in CEA and CA19-9 between the PNI-positive and PNI-negative groups.

Eight radiomics features related to PNI were selected for this study. Kulkarni et al. (36) extracted CT texture features to analyze their association with PNI and did not find any texture features related to PNI. The possible reason is that the texture features were only extracted from the maximum level of the tumor, which included incomplete features. In addition, in this study, poorly vascularized tumors located in the head of the pancreas were selected. In our study, the radiomics features of the whole lesion were extracted at the three-dimensional level, which could help to discover more biological characteristics of tumors. These selected features were integrated into a Rad-score and exhibited moderate performance in preoperatively predicting the PNI status of PDAC in both the training and testing cohorts. Radiomics can improve the prediction performance of medical images by improving analysis and using computer algorithms to extract thousands of quantitative features, and it can mine a large amount of information that is invisible to the naked eye. According to the radiologists’ evaluation, the CTPNI achieved inferior performance, which may be attributed to perivascular inflammation or fibrosis easily mimicking PNI on CT. In addition, PDAC is characterized by lymphatic growth and PNI, which are easily confused with microvessels, LN or fibrosis on CT. This may have caused the unsatisfactory agreement between the two reviewers in assessing the CTPNI and CTLN in our study.

A nomogram combining the Rad-score, CTLN and CTPNI achieved the best performance (the AUC in the testing cohort was 0.778) for the preoperative assessment of PNI in patients with PDAC. Several possible reasons may contribute to the good performance of the nomogram. One is that the Rad-score combined with arterial and portal venous phases can provide valuable information. In addition, different CT scan parameters may lead to unsatisfactory reproducibility of radiomics features, which can be maximally alleviated by using resampling as a preprocessing method, which optimizes gray dispersion to maintain the stability of features (37). Z score standardization eliminates the effect of different data dimensions. Moreover, the good performance of the nomogram was attributed to feature selection and modeling. Univariate analysis and LASSO regression confirmed that important features were selected for modeling. Tenfold validation was applied to guarantee the robustness of the model. Finally, the nomogram integrates selected radiomics features, the presence of PDAC on CT images and the experience of radiologists and combines the performance of different dimensions to better reflect the characteristics of PDAC. This result suggested that the nomogram has the potential to preoperatively predict PNI status in PDAC patients. The calibration curve revealed that the predicted PNI was in good agreement with the actual PNI probability. The decision curve indicated that the nomogram outperformed radiologists at any threshold probability.

This study has several limitations. First, this was a single-center study with a limited sample size and no external validation group. However, the number included in our study was relatively larger than that in previous studies. Therefore, the retrospective nature of the study may have led to biased results. Large sample, multicenter and prospective studies should be conducted to further verify the results. In addition, the study just delineated the tumor and extracted the radiomic features, which reflect the internal characteristics and biological behavior of the tumor. The space around the tumor was not sketched to extract additional features, which may be the reason for our modest results. The tumor and peritumoral radiomics features analysis need further study. Moreover, although several studies have reported that PNI is associated with PDAC patient prognosis, the relationship between PNI and prognosis was not clarified in this study. Subsequently, the corresponding patients should be followed up on the basis of this study to explore the ability of the nomogram to predict survival. Ultimately, there was not a consistent one-to-one match between CT evaluation and pathology.

In conclusion, a nomogram based on the rad-score derived from both the arterial and portal venous phases of CECT combined with the CTLN and CTPNI may serve as a valuable noninvasive tool for the preoperative assessment of PNI in patients with PDAC. This approach offers a practical means to classify PDAC patients before surgery and enhance patient management.

## Data Availability

The raw data supporting the conclusions of this article will be made available by the authors, without undue reservation.
