# Supplementary material for: A CT-based radiomics nomogram for the preoperative prediction of perineural invasion in pancreatic ductal adenocarcinoma
Source: Front Oncol. 2025 Mar 4;15:1525835. doi: 10.3389/fonc.2025.1525835 (PMC11913684; doi:10.3389/fonc.2025.1525835)
Supplement: Supplementary file 1 [file DataSheet1.docx]

**Supplementary material**

**Supplementary A1: CT scanning parameters and contrast agents.**

All patient underwent contrast-enhanced abdominal CT scan on Siemens Definition Dual-source CT at our institution. The scan ranges from the inferior chest to the pelvis or iliac fossa. The tube current ranged from 200 mA to 210 mA, and the tube voltage was 120kV. Collimation 0.625, pitch 0.8-1.0, thickness 2-5 mm. The nonionic contrast medium (Omnipaque 350; GE Healthcare, Chicago, IL) was administered intravenously at an injection rate of 2.5 -3.5ml/s. The arterial phase and portal vein phase were acquired by 30-35s and 60 to 70s after injecting contrast agent, respectively.

**Supplementary S1.** **Feature extraction**

Feature groups including gray level cooccurence matrix (GLCM), gray level runlength matrix (GLRLM), intensity histogram (IH), intensity direct (ID) and shape were extracted from IBEX. Feature explanation was supplemented in IBEX (β1.0, <http://bit.ly/IBEX_MDAnderson>, the University of Texas MD Anderson Cancer Center, Houston, USA). For the percentiles and percentile area of the IH, the calculation range is 5th percentile ~ 95th percentile (5% intervals). The 25th, 50th, 75th, and 95th quantiles were calculated; the remaining 6 features were calculated by one parameter. For the percentiles in ID, the calculation range is 5th percentile ~ 95th percentile (5% intervals). The 0.025, 0.25, 0.5, 0.75 and 0.975 quantiles were calculated; the remaining 30 features were calculated by one parameter. For the GLCM, four directions (*θ* = 0°, 45°, 90°, 135°) and three offsets (*d*= 1, 4, 7) were measured for a total of 264 generated features. For the GLRLM, three directions (*θ* = 0°, 90°) and one offset (*d*= 1) were measured for a total of 22 generated features. 16 features in the shape group were included. In total, 808 features were extracted from arterial phase and portal vein phase for each patient.

**Supplementary S2.** **Preprocessing methods for the image and data**

S2.1 Resampling

In our study, CT images were collected using variable acquisition techniques and parameters, which leading the radiomics features are difficult to reproduce. To diminish the influence of the variable CT parameters, we adopted resampling as a preprocessing method, which was performed to obtain a voxel size of 0.7 x 0.7 x 0.5 mm^3^ via trilinear interpolation before feature calculation.

S2.2 Z-score standardization

Different radiomics features have different ranges of values, and features of different magnitudes are difficult to compare. Before further analysis, we used z-score standardization to eliminate the effects of different dimensions by scaling the values to a mean of 0 and a standard deviation of 1 using the following formula:

z = $\frac{\chi-\mu}{\sigma}$

where *μσ* is the population [mean](https://en.wikipedia.org/wiki/Mean) and *σ* is the population [standard deviation](https://en.wikipedia.org/wiki/Standard_deviation).

**Supplementary S3. Intra-observer and inter-observer reproducibility evaluation**

In ore to evaluation of feature stability, the inter-observer and intrao-bserver agreement based on the interclass correlation coefficient (ICC) were applied. ICC score greater than 0.75 was considered to have good consistency. The result of the inter-observer and intrao-bserver agreement were shown in Supplementary Figure S1.

**Supplementary S4. Features for modeling**

After LASSO algorithm, X90.4Correlation, X90.7SumVariance.1, Orientation.1 X40Percentile.2, X10PercentileArea Energy.1, ConvexHullVolume3D.1 and MeanBreadth.1 in the arterial phase and portal vein phase were selected for modeling.

**Supplementary Figure S1: Intra-observer and inter-observer reproducibility evaluation.**


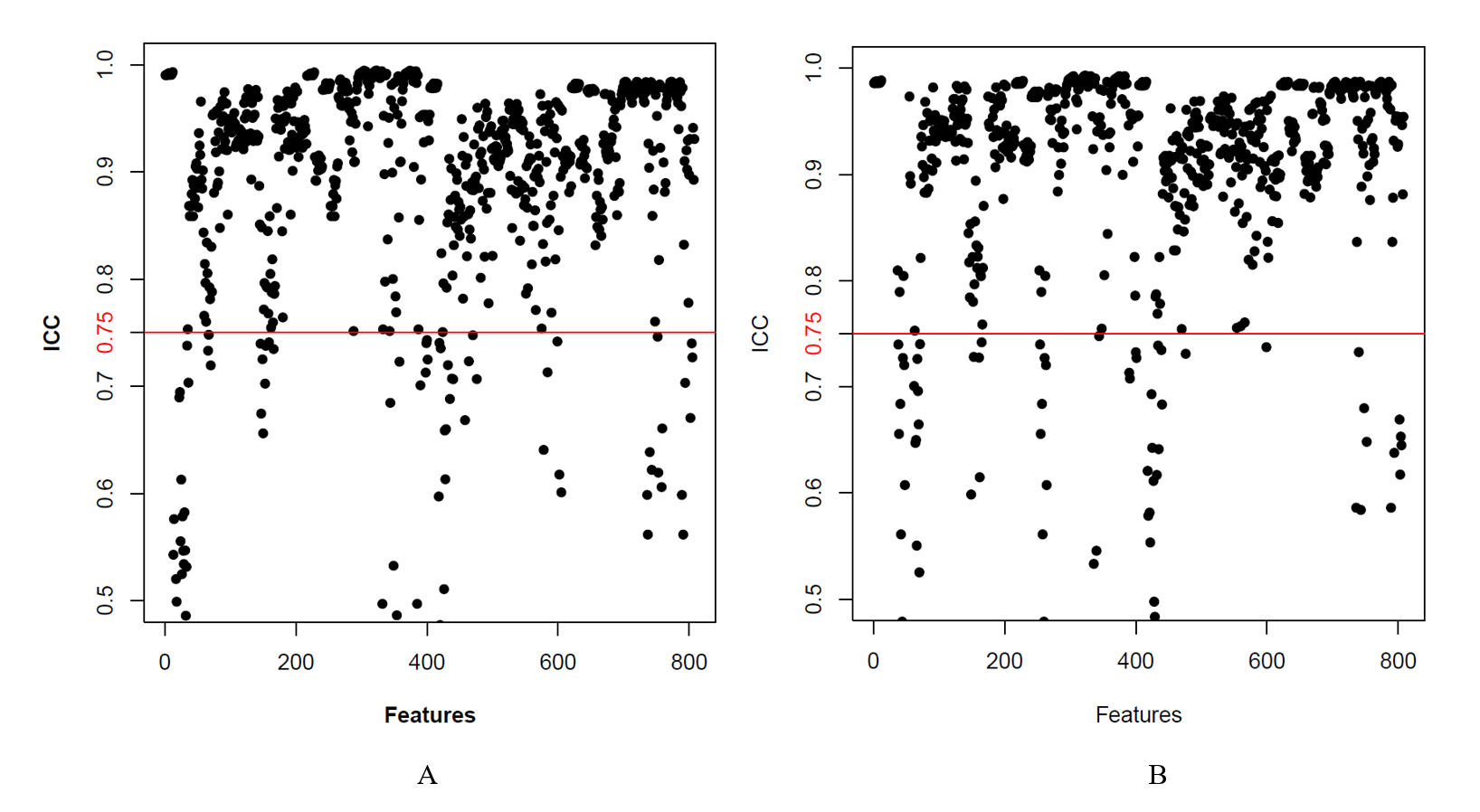


All features above the red cutoff line presented good intra-observer agreement with ICCs of > 0.75 (A) and good inter-observer agreement with ICCs of > 0.75 (B).
